# Complications of Neurofibromatosis 1 (NF1) in an Adult With Multiple Comorbidities

**DOI:** 10.7759/cureus.16512

**Published:** 2021-07-20

**Authors:** Ozioma P Nnomadim, Blandine Bustamante Helfrich

**Affiliations:** 1 Medicine, University of the Incarnate Word School of Osteopathic Medicine, San Antonio, USA; 2 Pathology and Faculty Affairs, University of the Incarnate Word School of Osteopathic Medicine, San Antonio, USA

**Keywords:** neurofibroma, depression, osteopathy, opioid-abuse, chronic pain

## Abstract

Neurofibromatosis (NF) is an autosomal genetic disorder with three types, including NF1, NF2, and schwannomatosis. It is characterized by bulging and deforming masses arising from multiple nerves involving skin folds and connective tissues. Prompt diagnosis and provision of care for NF1 patients by clinicians aware of the diverse clinical features of this disorder are needed for optimum patient care and management.

A 65-year-old African American female with a past medical history significant for multiple neurofibromas covering more than 95% of her total body surface area (TBSA), presented to a primary care clinic with an enlarged ulcerated neurofibroma of the right elbow. She reported associated pulsating, sharp pain, which was radiating to her entire right upper extremity. For most of her adult life, the lesion has been present and began as the rest of the neurofibromas on her body but gradually enlarged with eventual ulceration three months before the visit. The patient reported a failed surgical resection for the same neurofibroma several years ago. She also reported diffuse tenderness of the lesion, which severely impaired her daily living activities and limited her sleep ability. She acknowledged using multiple over-the-counter analgesics, prescription hydrocodone/acetaminophen 5/325 mg as needed, and gabapentin 300 mg orally twice daily but denied significant symptom alleviation. The patient was started on oral clindamycin hydrochloride 300 mg every six hours for 10 days and a topical mupirocin ointment 2% three times daily for five days. Subsequent visits showed no improvement of the ulcer, which necessitated a referral to wound care. After multiple wound care visits without progress, the patient was referred to a plastic surgeon for evaluation for repeat ulcer resection.

NF1 patients develop multiple tumors (neurofibromas); approximately 8%-15% percent of them present with malignant peripheral nerve sheath tumors (MPNST) within the patient’s lifetime. Tumor ulceration is a rare but possible complication of NF1. Due to the acute ulcerated fibromas’ complications, previous unsuccessful cosmetic management, and ambiguity about NF1 disorder, the patient’s quality of life was impaired. The physical and emotional pain the patient experienced impacted her activities of daily living and likely contributed to or exacerbated her diagnoses of substance use disorder and major depressive disorder.

NF1 is incurable and can be associated with complications that deteriorate the quality of life, depending on symptom severity. The condition impacts patients’ bodies, minds, and spirits, as seen in this patient who had diagnoses of substance use disorder and major depressive disorder, as well as a history of suicidal ideation and suicide attempts. The treatment of conditions related to NF1 is best managed in centers equipped with doctors experienced in treating patients with NF1. A multidisciplinary management approach is ideal. Preferably, for the management of chronic pain and beyond, the osteopathic holistic approach, targeting the body, mind, and spirit, in combination with other innovative non-pharmacotherapies and pharmacotherapy methods, would be beneficial.

## Introduction

Neurofibromatosis (NF) is of three distinct genetic disorders with the occurrence of tumors of the nerve sheath. They include NF1, NF2, and schwannomatosis [1-9]. They are dominantly inherited with a high rate of new mutations and variable expression. Multiple body system effects are primarily associated with NF1 with the tumor known as neurofibroma. Additionally, bone dysplasia, learning disabilities, and increased risk of malignancy are other known perils [4-5]. Due to the varying features and clinical assortment inherent to this disorder, patients can visit different medical and surgical experts. Thus, a multidisciplinary approach to care is strongly advocated with a dedicated team of specialists throughout the patient’s lifetime. A deepening understanding of this disorder through basic laboratory and clinical investigations, followed by the swift implementation of new, effective treatment, may facilitate the desirable and long-awaited breakthrough care for NF1 patients [10].

## Case presentation

A 65-year-old African American female, with a past medical history significant for multiple neurofibromas covering more than 95% of her total body surface area (TBSA), presented to a primary care clinic with an enlarged ulcerated neurofibroma of the right elbow. She reported associated pulsating, sharp pain, which was radiating to her entire right upper extremity. For most of her adult life, the lesion has been present and began as the rest of the neurofibromas on her body but gradually enlarged with eventual ulceration three months before the visit. The patient reported failed surgical resection for the same neurofibroma several years ago. She also reported diffuse tenderness of the lesion, which severely impaired her daily living activities and negatively impacted her sleep ability. She acknowledged using multiple over-the-counter analgesics, prescription hydrocodone/acetaminophen 5/325 mg as needed, and gabapentin 300 mg orally twice daily but denied significant symptom alleviation. The patient was started on oral clindamycin hydrochloride 300 mg every six hours for 10 days and a topical mupirocin ointment 2% three times daily for five days. Subsequent visits showed no improvement of the ulcer, which necessitated a referral to wound care. After multiple wound care visits without progress, the patient was referred to a plastic surgeon for evaluation for repeat ulcer resection.

Medical history

**Figure 1:**
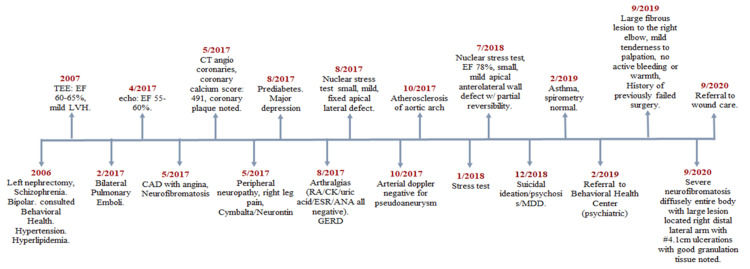
Timeline of available medical history

**Figure 2:**
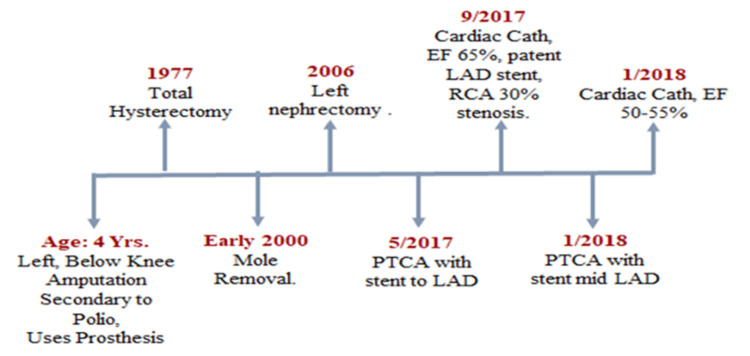
Timeline of available surgical history LAD: Left Anterior Descending; RCA: Right Coronary Artery; EF: Ejection Fraction; PTCA: Percutaneous Transluminal Coronary Angioplasty

**Figure 3:**
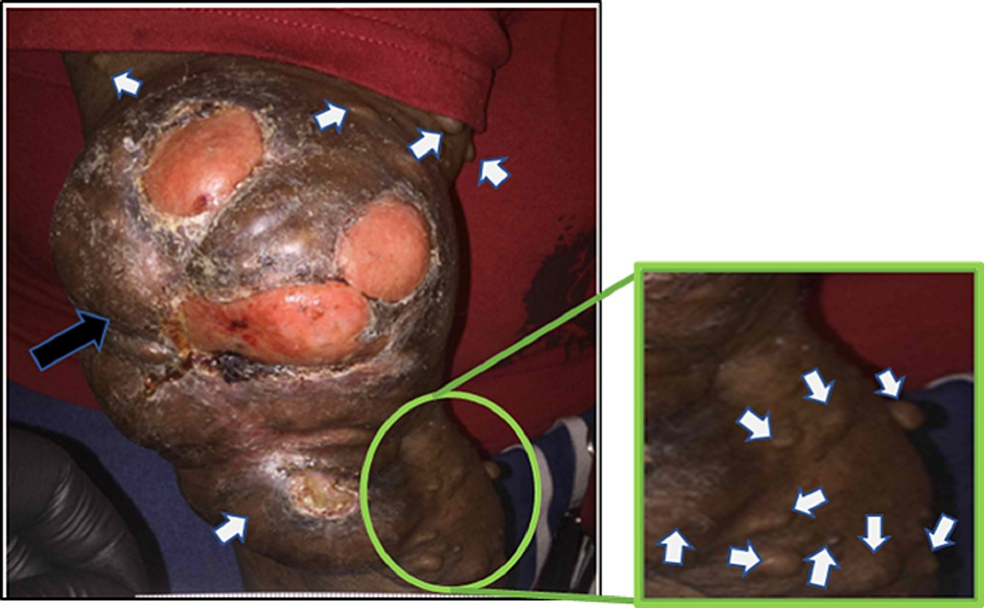
Ulcerated neurofibroma Neurofibroma over right elbow with significant stretching of the skin Black arrow: large neurofibroma with multiple open wounds (ulcer of the elbow with fat layer) White arrow: multiple neurofibromas

**Figure 4:**
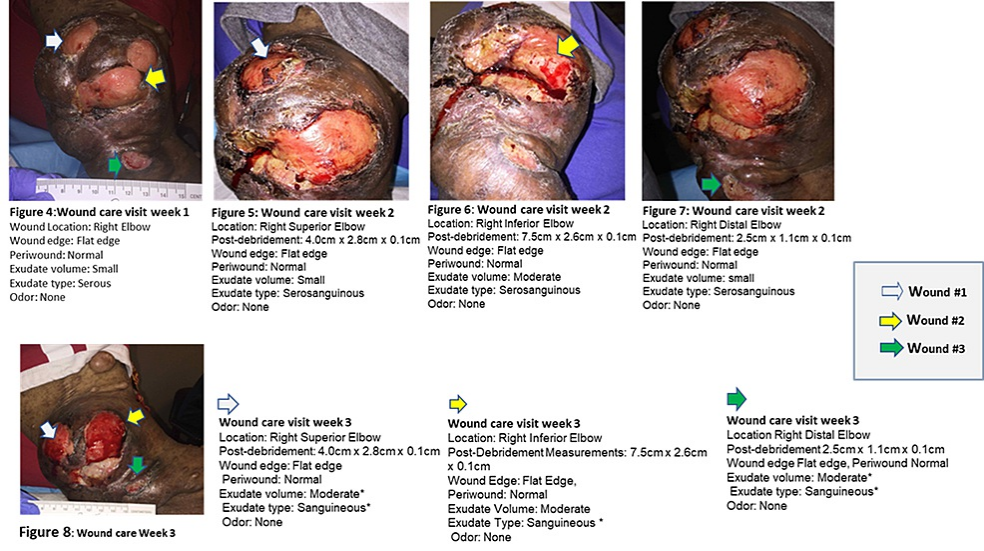
Wound care visits - week 1 to week 3 The treatment procedure encompasses a conservative bedside sharp debridement to subcutaneous tissue with a dermal curette, removing devitalized tissues, limited by the patient’s pain/sensitivity. Surgical management was advised from the start of therapy.

See Figures 1-2 for her medical and surgical history. Figure 3 shows the ulcerated neurofibroma of the right elbow while Figure 4 shows images from various wound care visits.

## Discussion

NF1 is a common hereditary disorder that affects about one in 4,000 births, regardless of gender, race, or ethnic origin [4,7,10-11]. It is a chronic multisystem disorder that targets several different tissues [6]. NF is an autosomal dominant disorder located on one of the 22 chromosome pairs. NF1 is located on chromosome 17 while NF2 is located on chromosome 22 [6]. The heterozygous pathogenic variants in NF1, accountable for type 1 neurofibromatosis as present in this patient, may lead to several developmental anomalies and an increased risk of learning disabilities.

The disabilities associated with NF1 affect all genders equally, and the occurrences are seen among all ethnic groups [6]. NF1 symptoms differ per individual and include freckles in the groin or axilla, scoliosis (severe deformation or curvature of the spine), presence of six or more café-au-lait spots (light-brown macules on the skin), and multiple pea-sized cutaneous or subcutaneous nodules (neurofibromas) associated with nerve tissues [2,7- 8]. Additional manifestations include Lisch nodules (tiny tan clumps of pigment in the iris of the eyes), optic glioma (a tumor along the eye’s optic nerve), and bone malformations [2,7-8].

The diagnosis of NF requires physical examination done by medical practitioners conversant with NF disorder. For children, at least two or more symptoms associated with NF1 must be present to make the diagnosis. A combination of findings is required when diagnosing NF [3], and it is usually based on clinical findings. The patient, in this case, was diagnosed years ago and must have gone through several examinations and, perhaps, different consultations with various medical experts before a conclusive diagnosis was reached.

Among many clinical findings to ascertain NF diagnosis, skin examination for café-au-lait spots is one of the prominent cues to further investigate possible NF involvement. However, the skin assessment may require a Wood’s lamp [3]. Wood’s lamp for skin assessment is helpful in fair-skinned persons for a more accurate examination of the skin for a precise diagnosis. CT scans, MRIs, and X-rays may be used to confirm or rule out scoliosis and other possible bone or internal deformations [3].

The advent of innovations in genomic testing ensures that blood tests may be relied on to detect defects in the NF1 gene. Genomic testing has solidified mutation analysis, which is 95% accurate in discovering NF1 mutation [7]. The pervasive use of these testing lines has become ubiquitous in diagnosing NF disorder and other medical conditions that were once painstaking to diagnose and treat. Linkage testing is another genetic test that is approximately 90% accurate in determining if individuals have NF. Although rarely needed to diagnose NF1, molecular genetic testing could be helpful [3,7]. Another option is cytogenetic testing, which is typically used if a clinical diagnosis of NF1 is made but no pathogenic changes of NF1 cDNA (mRNA), gDNA, and copy number are detected [3]. It should be noted that in fewer than 1% of NF1 affected individuals, cytogenetic rearrangements are responsible for the NF1 disorder [3].

Currently, NF1 is not curable but can be managed by multiple specialties. An interdisciplinary team that includes optometrists, ophthalmologists, neurologists, cardiologists, endocrinologists, surgeons, orthopedics, and dermatologists is best able to provide supportive care for patients with NF1 [8]. The disfiguring and sometimes uncomfortable, discrete, cutaneous, or subcutaneous neurofibromas can be surgically removed. Dystrophic scoliosis often requires surgical management, whereas non-dystrophic scoliosis can usually be treated conservatively [12]. Although the patient, in this case, has scoliosis, she did not undergo surgical correction for it. However, the patient underwent amputation of her lower limb at a young age related to polio. While unrelated to her NF1 diagnosis, the procedure further exacerbated her disability as she has aged.

The patient presented with hundreds of neurofibromas all over her body, ranging in various sizes. Among many other chronic multisystem NF disorders, NF1 patients develop multiple tumors (neurofibromas) [2-3,7-8,9,13]. Approximately, 8%-15% of them present with malignant peripheral nerve sheath tumor (MPNST) at some point in life [6]. The associated complications with NF1 negatively impact patients’ quality of life and underpin the disease’s ambiguity [1].

This genetic disorder has no cure, only symptom management [8]. However, surgical resections of fibromas are performed in particular situations for cosmetic and other medical reasons. Often, surgical treatment of plexiform neurofibromas has shown to be unsatisfactory. However, when possible, complete surgical excision of malignant peripheral nerve sheath tumors is an option [7,14]. The patient had a large lesion removed from her right elbow several years ago, but the lesion regenerated with constant, excruciating pain. The investigation to rule-in or rule-out plexiform in this patient was discussed but not explored due to treatment interruption from multiple specialty consults, visits, and the patient’s unfortunate premature demise.

Chronic pain is an issue with certain patients with NF1, which decreases their quality of life and productivity [3]. The patient was prescribed pain medications that did not seem to mitigate or alleviate her pains. The patient complained of continued excruciating pain in the right elbow. The patient confirmed pain of 9/10 on a numeric rating scale of 0-10 with the movement of the right elbow. On examination, she was unable to extend or flex the arm fully. She was unable to supinate or dorsiflex her wrist due to extreme pain. This significantly impaired her daily functioning, particularly because the patient was right-handed. The patient could not do basic housekeeping chores, shop for her groceries, or prepare her food. She acknowledged using multiple over-the-counter analgesics, prescription hydrocodone/acetaminophen 5/325 mg as needed, and gabapentin orally, twice daily, but denied significant symptom alleviation. Her continued debilitating pain prompted increases in her prescription pain medication dosages, in an attempt at better pain control.

The multiple tissue involvement, nerve damage associated with NF, and this patient’s ulcerated neurofibromas caused excruciating pain. This pain was disabling for the patient and led her to seek out measures to mitigate or alleviate the discomfort [15]. The patient’s quest for comfort led her to several medical specialty consults, which resulted in numerous pharmacological prescriptions, including hydrocodone. Physicians often prescribe opioids, and that contributes to opioid overdose and dependence, especially with prolonged use [16].

The association of chronic pain with psychiatric disorders, predominantly anxiety and depression, is known, although often overlooked, in clinical practice [12]. This patient had several episodes of suicidal ideations and a diagnosis of major depressive disorder (MDD), resulting in several psychiatric referrals. Osteopathic medicine’s holistic approach to treat body, mind, and spirit should be considered by interdisciplinary teams who provide care to patients with chronic pain and other NF1-related symptoms.

A random clinical trial (RCT) found that patients receiving spinal manipulative therapy from an osteopathic physician to treat lower back pain correlated positively to changes in pain sensitivity and anxiety [17]. Another practical trial conducted by Williams demonstrated that osteopathic manipulative treatment (OMT) provided by an osteopathic physician enhanced the Short Form 12 (SF-12) measure’s mental health score [18]. Williams later urged the integration of cognitive-behavioral principles to optimize the psychological benefits of spinal manipulation [19]. Although OMT was not performed on this patient, its exploration or trial before commencing pharmacotherapy or combination with conventional therapy might have delayed, at least temporarily, some of her behavioral reactions to chronic pain [16]. Jerome posited that nonopioid therapies are preferred for managing chronic pain other than that associated with active cancer and palliative and end-of-life care. Jerome further stated that cognitive-behavioral therapy, in combination with physical strategies, such as exercise and osteopathic manipulative medicine (OMM), can be frontline strategies for chronic pain management [16].

Also, in an RCT studying the use of OMT to treat patients with fibromyalgia-related chronic pain, significant differences were shown in the Beck Depression Inventory (BDI) scores between experimental and control groups after a one-year follow-up [20]. These non-pharmacological therapy modalities are worth exploring and incorporating into the care of NF1 patients [20]. The above assertion, encouraging the incorporation of non-pharmacological therapy modalities, supports the US Surgeon General’s suggestion of non-pharmacologic management for patients with chronic pain. OMT, as part of a comprehensive management plan, can help reduce the risk of substance dependence by providing a more holistic approach to managing chronic pain. [16]. Given the mental health impact of diseases with multiple comorbidities and chronic pain, OMT should be incorporated in inpatient care. Self-reported outcomes in a prospective observational study by Edwards and Toutt of patients with chronic lower back pain showed that OMT improved self-care and diminished pain and anxiety (although not depression) [12], OMT is a field of medicine that is underutilized yet holds great promise [12].

## Conclusions

The extensive effects of multiple tissue involvement associated with NF1 overwhelm many patients. Treatment by different medical experts can be discouraging, as patients are aware that none of their medical visits and numerous consultations will result in a cure. Most NF1 patients live with emotional or physical pain. They often become reclusive, depressed, and even attempt suicide to escape the dilemmas inherent with this chronic genetic disease. Physicians frequently prescribe opioids to patients with this condition in an attempt to treat their pain, but this results all too often in addiction and overdose. Therefore, a more holistic and alternative approach to pain management, such as OMT, should be considered to complement conventional medical care and possibly alleviate addictions and overdose. Hopefully, the innovative advancement in genetics will spur research interest in and new treatment modalities for NF1, bringing hope to current and future patients.
